# Association Between Breast Arterial Calcification on Mammography and Carotid Artery Stenosis on CT Angiography: A Retrospective Study in a Selected Cohort of Women Aged 60–65 Years

**DOI:** 10.3390/jcm15207844

**Published:** 2026-10-12

**Authors:** Laçin Tatlı Ayhan, Mehmet Ali Durmuş, Mehmet Oğuzman, Kıyasettin Asil, Yasemin Gündüz

**Affiliations:** 1 Department of Radiology, Faculty of Medicine, Sakarya University, 54290 Sakarya, Türkiye; dryasemingunduz@yahoo.com; 2 Department of Radiology, Sakarya Training and Research Hospital, 54100 Sakarya, Türkiye; m.alidurmus0@gmail.com (M.A.D.); mehmet.oguzman@saglik.gov.tr (M.O.); drkiyasettin@gmail.com (K.A.)

**Keywords:** breast arterial calcification, carotid stenosis, mammography, computed tomography angiography, atherosclerosis, postmenopausal women

## Abstract

**Background/Objectives:** Breast arterial calcification (BAC) is an incidental mammographic finding that may be associated with systemic vascular disease. We investigated the association between mammographic BAC and carotid artery stenosis on cervical computed tomography angiography (CTA) in a clinically selected cohort of postmenopausal women aged 60–65 years. **Methods:** This retrospective, single-center study screened 3421 women aged 60–65 years who underwent both mammography and cervical CTA between November 2021 and May 2025. CTA examinations requested from the emergency department or cardiology clinics, examinations not performed within the same calendar year, and prespecified clinical exclusions were removed, leaving 180 women who underwent outpatient CTA for nonspecific symptoms. The median interval between examinations was 131 days. BAC was graded on a 0–3 scale, and carotid stenosis was assessed according to the North American Symptomatic Carotid Endarterectomy Trial (NASCET) criteria, using the greater degree of stenosis on either side as the patient-level value. BAC was analyzed as an ordinal variable, as a categorical variable, and using binary definitions of presence and severity. Multivariable models were adjusted for age, diabetes mellitus, hyperlipidemia, hypertension, and smoking. **Results:** Clinically significant stenosis (≥50%) was present in 44 women (24.4%). Its prevalence increased from 5.4% in BAC grade 0 to 75.0% in grade 3 (*p* for trend < 0.001). Each one-grade increase in BAC was associated with clinically significant stenosis (adjusted odds ratio [aOR], 4.23; 95% confidence interval [CI], 2.50–8.03; *p* < 0.001). The corresponding aORs were 16.70 (95% CI, 4.99–73.27) for any BAC and 18.49 (95% CI, 5.93–70.46) for BAC grade ≥2. Adding BAC to the clinical model increased the area under the receiver operating characteristic curve (AUC) from 0.90 to 0.96 (DeLong *p* = 0.005); the optimism-corrected AUC was 0.95. Inter-reader agreement for BAC grading was substantial (weighted κ = 0.79). **Conclusions:** In this selected cohort of women aged 60–65 years who underwent clinically indicated CTA, BAC was associated with carotid artery stenosis independently of conventional cardiovascular risk factors. These findings do not establish BAC as a screening or prediction tool and require prospective, population-based validation.

## 1. Introduction

Breast arterial calcification (BAC) is an incidental finding on screening mammography. Although traditionally regarded as benign, recent studies suggest that BAC may be associated with systemic vascular disease, including coronary and carotid vascular disease [1,2]. Histologically, BAC is related to Mönckeberg medial sclerosis and has been associated with arterial stiffness and cardiovascular outcomes [3].

Several studies have examined the relationship between BAC and carotid atherosclerosis, including carotid intima–media thickness (IMT) and plaque formation [1]. The reported strength of this association, however, has been inconsistent. Age is an important potential confounder because both BAC and carotid atherosclerosis become more prevalent with advancing age [4]. Diabetes mellitus, hyperlipidemia, hypertension, and smoking may also contribute to vascular calcification and carotid atherosclerosis [2].

BAC may be clinically relevant because it can be identified on routine mammography without additional imaging or radiation. Nevertheless, BAC and carotid atherosclerosis are histologically distinct processes. BAC predominantly reflects calcification of the tunica media of small- and medium-sized muscular arteries (Mönckeberg-type medial sclerosis), whereas carotid stenosis generally results from intimal atherosclerotic plaque [5,6]. An association between these processes may therefore reflect shared cardiometabolic risk factors, vascular aging, or a systemic propensity to calcification rather than a common pathology [7,8]. Most previous studies have focused on carotid IMT or plaque rather than directly measured luminal stenosis on computed tomography angiography (CTA) [1,9], and it remains unclear whether such an association persists after adjustment for conventional cardiovascular risk factors.

Accordingly, this study aimed to examine the association of the presence and severity of mammographic BAC with carotid artery stenosis assessed by cervical CTA in a clinically selected cohort of postmenopausal women aged 60–65 years, and to determine whether this association persisted after adjustment for conventional cardiovascular risk factors. The narrow age range was chosen to reduce age-related confounding. We hypothesized that higher BAC grades would be associated with a higher prevalence of clinically significant carotid stenosis.

## 2. Materials and Methods

### 2.1. Study Design and Population

This retrospective, single-center study was approved by the Ethics Committee of Sakarya University Faculty of Medicine (Protocol No. 2025/583). The requirement for informed consent was waived in accordance with institutional policies and the Declaration of Helsinki.

Age is an important determinant of vascular calcification and may confound the association between BAC and carotid stenosis. To reduce this effect, we focused on women aged 60–65 years. Between November 2021 and May 2025, 16,895 women underwent mammography at our institution. Of these, 3421 were aged 60–65 years and also underwent cervical CTA during the study period. We excluded 2128 women whose CTA had been requested from the emergency department or cardiology clinics, leaving 1293 outpatient examinations. A further 1035 women were excluded because mammography and CTA had not been performed within the same calendar year. Among the remaining 258 women, exclusions were applied for malignancy (*n* = 20), reduction mammoplasty (*n* = 17), chronic kidney disease (*n* = 16), breast implants (*n* = 10), previous vascular intervention (*n* = 10), and breast trauma (*n* = 5), resulting in a final cohort of 180 postmenopausal women.

Women with a history of breast surgery, breast implants, or breast trauma were excluded because these factors could affect BAC assessment or image quality. Chronic kidney disease, known malignancy, and previous vascular intervention were excluded because of their potential effects on vascular calcification or vascular anatomy.

All CTA examinations had been requested from otorhinolaryngology or neurology outpatient clinics for evaluation of nonspecific symptoms, primarily dizziness/vertigo, tinnitus, or syncope. None of the included women had a documented stroke or transient ischemic attack in the hospital records. The order of the two examinations varied, and the median interval between mammography and CTA was 131 days (interquartile range [IQR], 75–217; range, 23–326 days). Thus, the study represents a clinically selected referral cohort rather than a population-based screening cohort and was designed as an opportunistic association study within this cohort.

### 2.2. Mammography and BAC Assessment

All mammograms were acquired in standard craniocaudal and mediolateral oblique projections using a Hestia mammography unit (Genoray Co., Ltd., Seongnam, Republic of Korea). A breast radiologist with 12 years of experience, blinded to the patients’ clinical history and CTA findings, reviewed the images on 5-megapixel diagnostic monitors (WIDE Corporation, Hwaseong, Republic of Korea).

**Figure 1:**
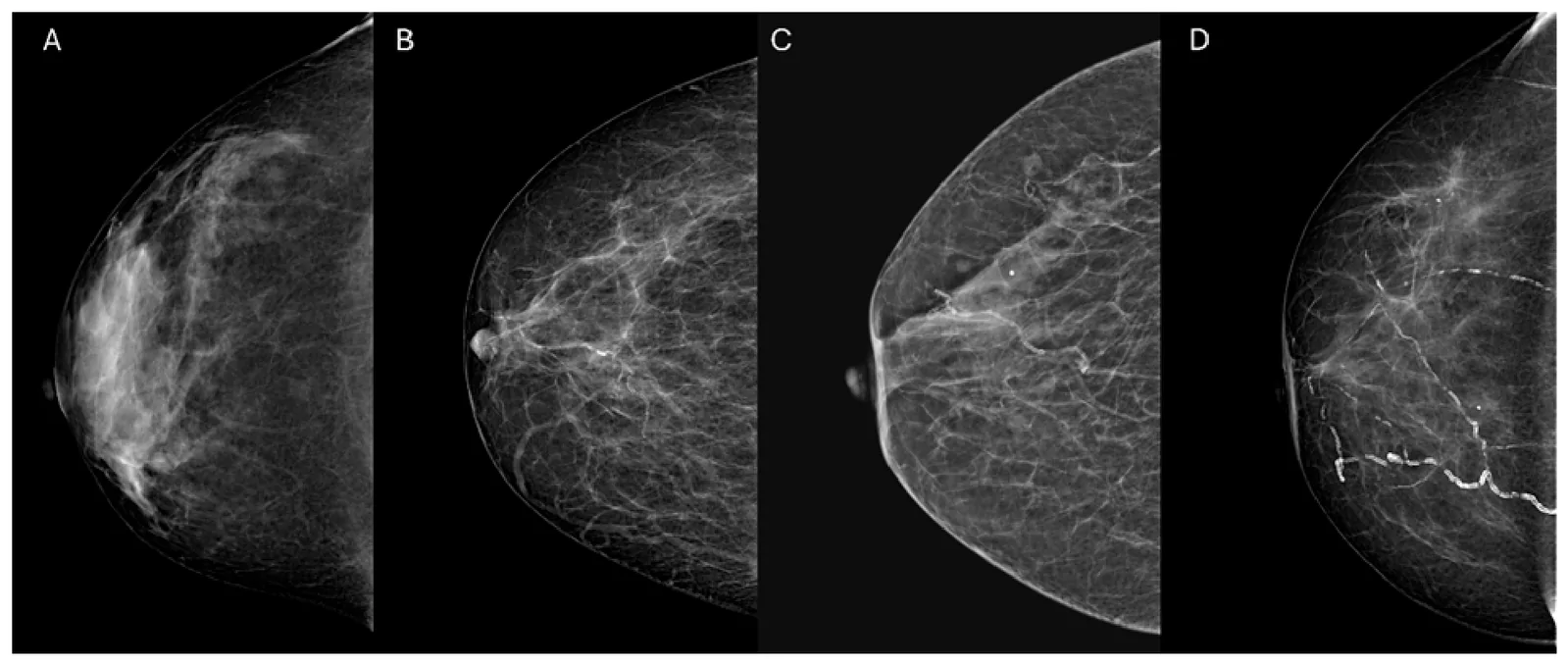
Representative craniocaudal mammographic images showing increasing BAC severity. (**A**) No calcification (grade 0). (**B**) Mild calcification (grade 1). (**C**) Moderate calcification (grade 2). (**D**) Severe calcification (grade 3), with a typical “railroad-track” appearance. BAC, breast arterial calcification.

BAC was defined as linear or ring-like calcification following the course of the arterial wall. Severity was graded using a four-point visual scale described in the literature [1]: grade 0, no vascular calcification; grade 1 (mild), sparse punctate or short linear calcifications; grade 2 (moderate), continuous linear calcification along the vessel; and grade 3 (severe), coarse calcification completely encircling or obscuring the vessel lumen (Figure 1). For patient-level analyses, the higher BAC grade of the two breasts was used.

Inter-reader reproducibility was assessed in a computer-generated random sample of 60 women from the study cohort, stratified by BAC grade (15 per grade). The original BAC assessment had been performed by the breast radiologist with 12 years of experience. Approximately 8 months later, the mammograms of these women were independently re-graded by the radiologist with 16 years of experience who had originally evaluated the CTA examinations. The cases were presented as a coded list in random order, and the second reader did not have access to the original BAC grades, the CTA findings, or the clinical data. Agreement between the two readers was assessed using weighted kappa (κ) statistics.

### 2.3. CT Angiography and Carotid Stenosis Assessment

Carotid artery stenosis was assessed on cervical CTA images acquired with a 64-slice CT scanner (Aquilion 64, Toshiba Medical Systems, Otawara, Japan). CTA was performed according to the institutional standard protocol after intravenous administration of 80 mL of iodinated contrast material (iopromide, Ultravist 300, Bayer HealthCare, Berlin, Germany) at 4 mL/s.

**Figure 2:**
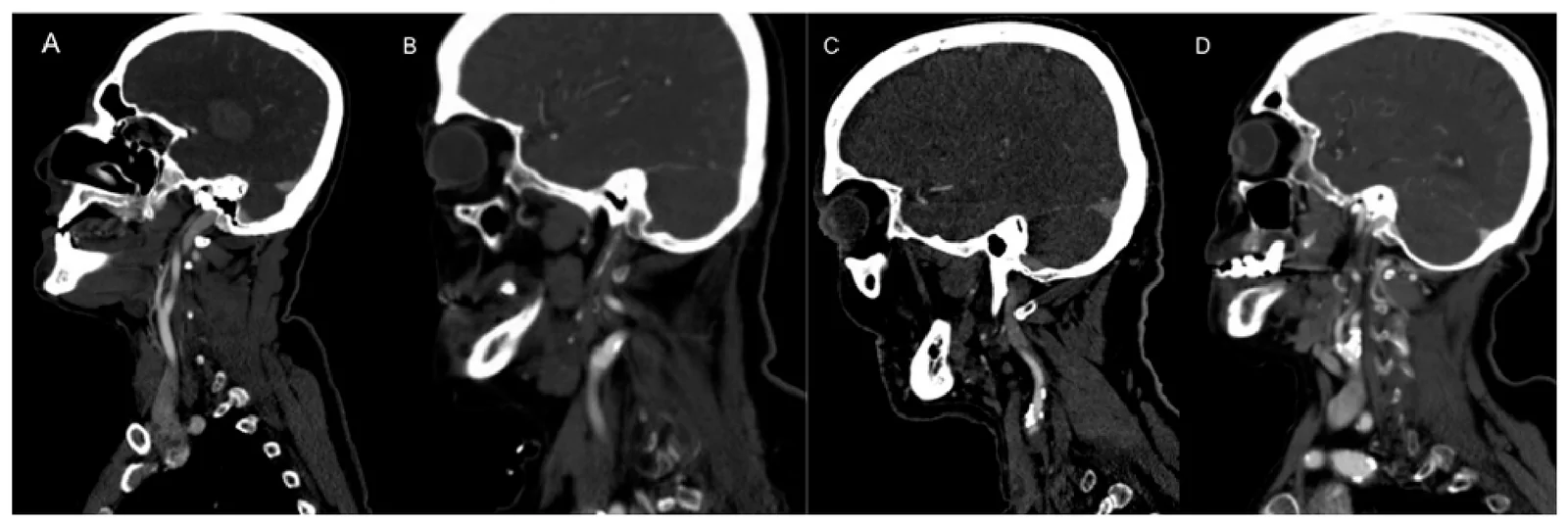
Sagittal reformatted maximum-intensity-projection images from cervical CT angiography showing representative examples of internal carotid artery stenosis. (**A**) Normal carotid artery. (**B**) Mild stenosis (<50%). (**C**) Moderate stenosis (50–69%). (**D**) Severe stenosis (≥70%).

CTA examinations were evaluated by a radiologist with 16 years of experience who was blinded to the mammographic BAC findings. Luminal stenosis was measured according to the North American Symptomatic Carotid Endarterectomy Trial (NASCET) criteria [10] using the formula (1 − [minimal residual lumen diameter at the stenosis/normal distal internal carotid artery diameter]) × 100. Measurements were obtained at the proximal internal carotid artery, including the carotid bulb, on multiplanar reformatted images perpendicular to the vessel axis; the reference diameter was measured at the first normal-appearing segment of the distal cervical internal carotid artery beyond the bulb, where the arterial walls are parallel. The extracranial internal carotid artery was assessed bilaterally, and the greater degree of stenosis was used as the patient-level value. Stenosis was categorized as grade 0, no stenosis; grade 1, mild (<50%); grade 2, moderate (50–69%); and grade 3, severe (≥70%) (Figure 2). Clinically significant stenosis was defined as ≥50%. One near-occlusion was classified as grade 3; no total occlusions were present.

### 2.4. Clinical Variables

Clinical data were obtained from the hospital information system using records closest to the imaging examinations. Diabetes mellitus was defined as fasting blood glucose ≥126 mg/dL or use of antidiabetic medication. Hyperlipidemia was defined as total cholesterol >200 mg/dL or use of lipid-lowering therapy [1,3,9]. Hypertension and smoking status were obtained from the medical records.

### 2.5. Statistical Analysis

Continuous variables are presented as mean ± standard deviation or median (IQR), and categorical variables as number and percentage. Women with and without clinically significant stenosis were compared using the Mann–Whitney U test and the chi-square or Fisher’s exact test, as appropriate; standardized mean differences were also reported as effect-size measures. The primary outcome was clinically significant carotid stenosis (≥50%), and severe stenosis (≥70%) was examined as a secondary outcome. Grade-specific proportions were reported with Wilson 95% confidence intervals (CIs). Trends across BAC grades were assessed using the Cochran–Armitage trend test, and the association between BAC grade and stenosis grade was evaluated using Spearman correlation.

To avoid excessive dichotomization, BAC was modeled in three complementary ways: as an ordinal variable (odds ratio [OR] per grade), as a categorical variable with grade 0 as the reference, and using the binary definitions from the original analysis (any BAC, grades 1–3 vs. grade 0; BAC severity, grades 2–3 vs. grades 0–1). Departure from linearity for BAC grade was assessed with a likelihood-ratio test comparing ordinal and categorical specifications. Four-level stenosis grade was additionally analyzed using proportional-odds ordinal logistic regression. Logistic regression models were fitted without adjustment, with adjustment for diabetes mellitus and hyperlipidemia (Model 1), and with adjustment for age, diabetes mellitus, hyperlipidemia, hypertension, and smoking (Model 2). Profile-likelihood 95% CIs and likelihood-ratio *p* values were reported. Firth penalized logistic regression [11] was used for sparse categorical analyses, severe-stenosis models, and selected sensitivity analyses.

Receiver operating characteristic (ROC) analysis was used to evaluate discrimination for clinically significant carotid stenosis by BAC grade, clinical variables, and BAC added to the clinical variables. Areas under the curve (AUCs) were reported with DeLong 95% CIs, and nested models were compared using paired DeLong tests [12]. Internal validation was performed with 1000 bootstrap resamples to estimate optimism-corrected AUCs. With 44 primary outcome events, Model 2 contained approximately seven events per parameter; the discrimination analyses were therefore considered exploratory rather than evidence of a validated prediction or screening model.

The effect of the interval between examinations was examined by adding the interval to Model 2, testing a BAC × interval interaction, and restricting analyses to women whose examinations were performed within 180 and 90 days. Inter-reader agreement for BAC grading was quantified using unweighted and linearly weighted kappa statistics with bootstrap 95% CIs and interpreted according to Landis and Koch [13]. All tests were two-sided, and *p* < 0.05 was considered statistically significant. Statistical analyses were performed using IBM SPSS Statistics version 26 (IBM Corp., Armonk, NY, USA) and Python 3.11 (Python Software Foundation, Wilmington, DE, USA) with SciPy 1.17 (https://scipy.org).

## 3. Results

**Figure 3:**
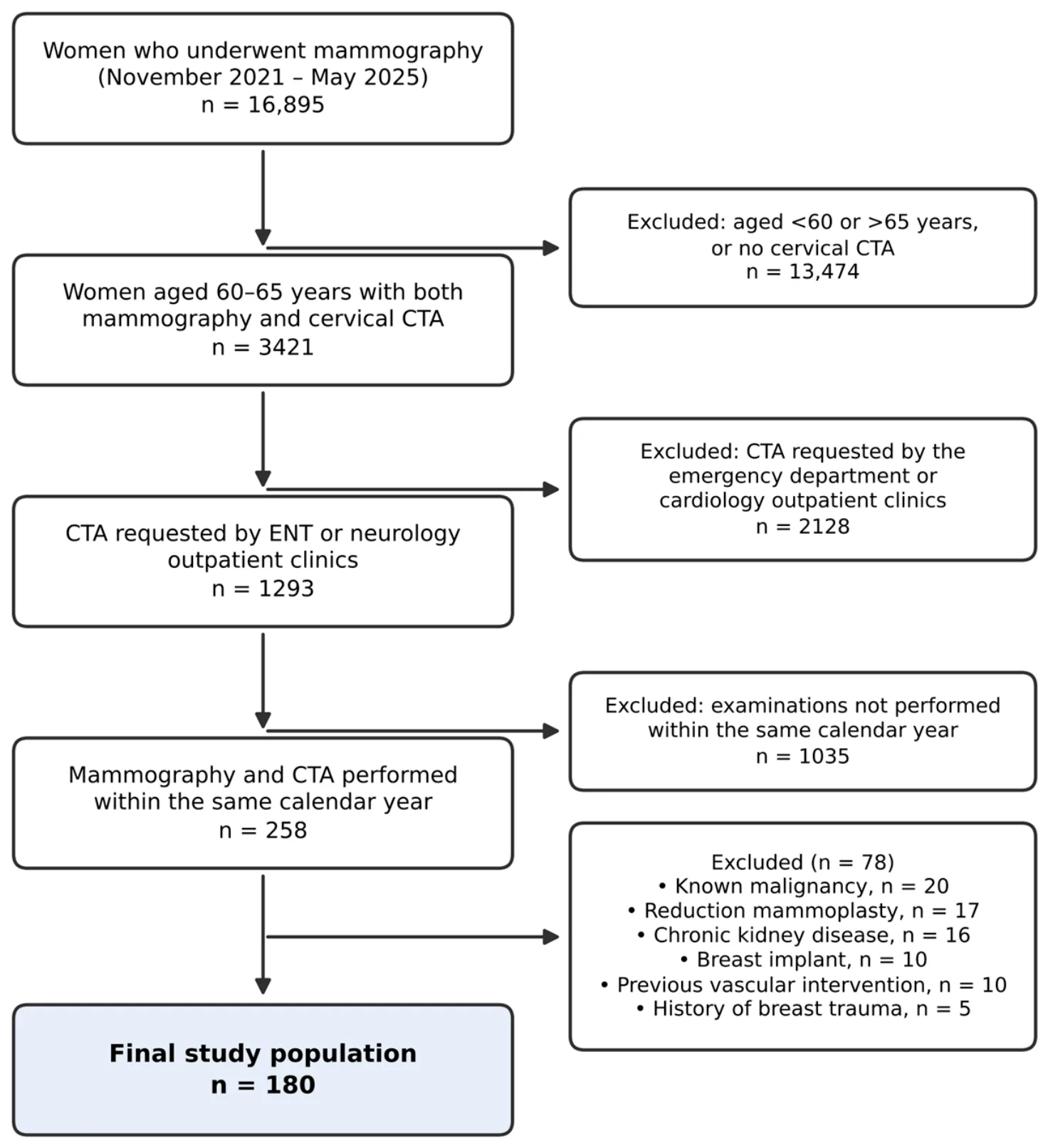
Flow diagram of patient selection from the initial mammography database to the final study cohort. CTA, computed tomography angiography; ENT, otorhinolaryngology.

The selection of the study population is shown in Figure 3. The final cohort comprised 180 women with a mean age of 62.1 ± 1.8 years. BAC was present in 87 women (48.3%): grade 1 in 27 (15.0%), grade 2 in 32 (17.8%), and grade 3 in 28 (15.6%). Regarding carotid stenosis, 48.9% had no stenosis, 26.7% had mild stenosis, 13.9% had moderate stenosis, and 10.6% had severe stenosis. Clinically significant stenosis was present in 44 women (24.4%). Women with clinically significant stenosis were older and more frequently had diabetes mellitus, hyperlipidemia, hypertension, and a history of smoking; the interval between examinations did not differ between groups (Table 1). Characteristics according to BAC grade are presented in Table S6.

**Figure 4:**
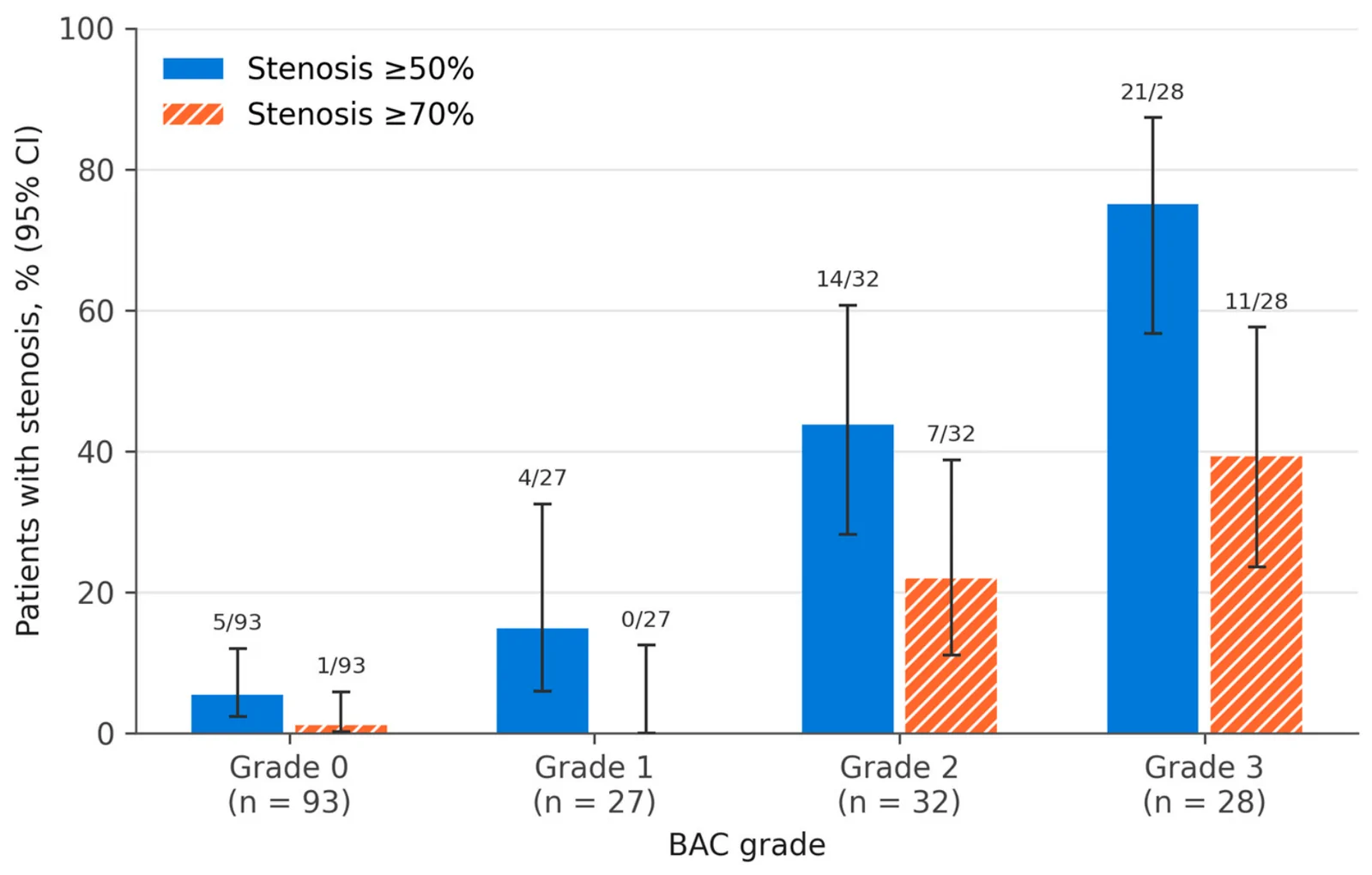
Proportions of women with clinically significant (≥50%) and severe (≥70%) carotid stenosis according to BAC grade (*n* = 180). Error bars represent Wilson 95% confidence intervals; numbers above the bars indicate events/total. *p* for trend < 0.001 for both outcomes.

As shown in Table 2 and Figure 4, the proportion of women with clinically significant stenosis increased from 5.4% (95% CI, 2.3–12.0) in BAC grade 0 to 14.8% (5.9–32.5) in grade 1, 43.8% (28.2–60.7) in grade 2, and 75.0% (56.6–87.3) in grade 3 (*p* for trend <0.001). BAC grade correlated moderately with stenosis grade (Spearman ρ = 0.66; *p* < 0.001). Grade 1 showed only a modest increase over grade 0, with no cases of severe stenosis (0/27; 95% CI, 0–12.5%); the difference between grades 0 and 1 was not statistically significant. The corresponding 2 × 2 tables for the binary BAC definitions are provided in Table S1.

In logistic regression analysis (Table 3), each one-grade increase in BAC was associated with clinically significant stenosis both before adjustment (OR, 3.81; 95% CI, 2.63–5.85) and after full adjustment for age, diabetes mellitus, hyperlipidemia, hypertension, and smoking (aOR, 4.23; 95% CI, 2.50–8.03; *p* < 0.001). In the fully adjusted categorical model, the aORs relative to grade 0 were 3.58 (95% CI, 0.65–20.20; *p* = 0.125) for grade 1, 10.55 (2.74–49.40; *p* < 0.001) for grade 2, and 49.68 (11.25–294.27; *p* < 0.001) for grade 3. There was no evidence of departure from a linear effect across BAC grades (*p* = 0.91). The fully adjusted aOR was 16.70 (95% CI, 4.99–73.27) for any BAC and 18.49 (5.93–70.46) for BAC grades 2–3. In the ordinal model using four-level stenosis grade as the outcome, each increase in BAC grade was associated with greater stenosis severity (aOR, 3.71; 95% CI, 2.69–5.14; *p* < 0.001).

In the fully adjusted model, hyperlipidemia, hypertension, smoking, and age were also independently associated with clinically significant stenosis, whereas the association with diabetes mellitus did not reach statistical significance (*p* = 0.061) (Table S2). BAC grade was also associated with severe stenosis (aOR per grade, 2.68; 95% CI, 1.52–5.47; *p* < 0.001) (Table S3). The interval between examinations was not correlated with BAC grade (ρ = 0.02; *p* = 0.81) or stenosis grade (ρ = −0.03; *p* = 0.70), and adding it to Model 2 produced a similar BAC estimate (aOR, 4.29; 95% CI, 2.52–8.28). There was no evidence of a BAC × interval interaction (*p* = 0.18). Results were also consistent when analyses were restricted to examinations performed within 180 days (*n* = 109; aOR, 4.68; 95% CI, 2.42–11.78) or 90 days (*n* = 59; aOR, 3.32; 95% CI, 1.52–10.63) (Table S4).

**Figure 5:**
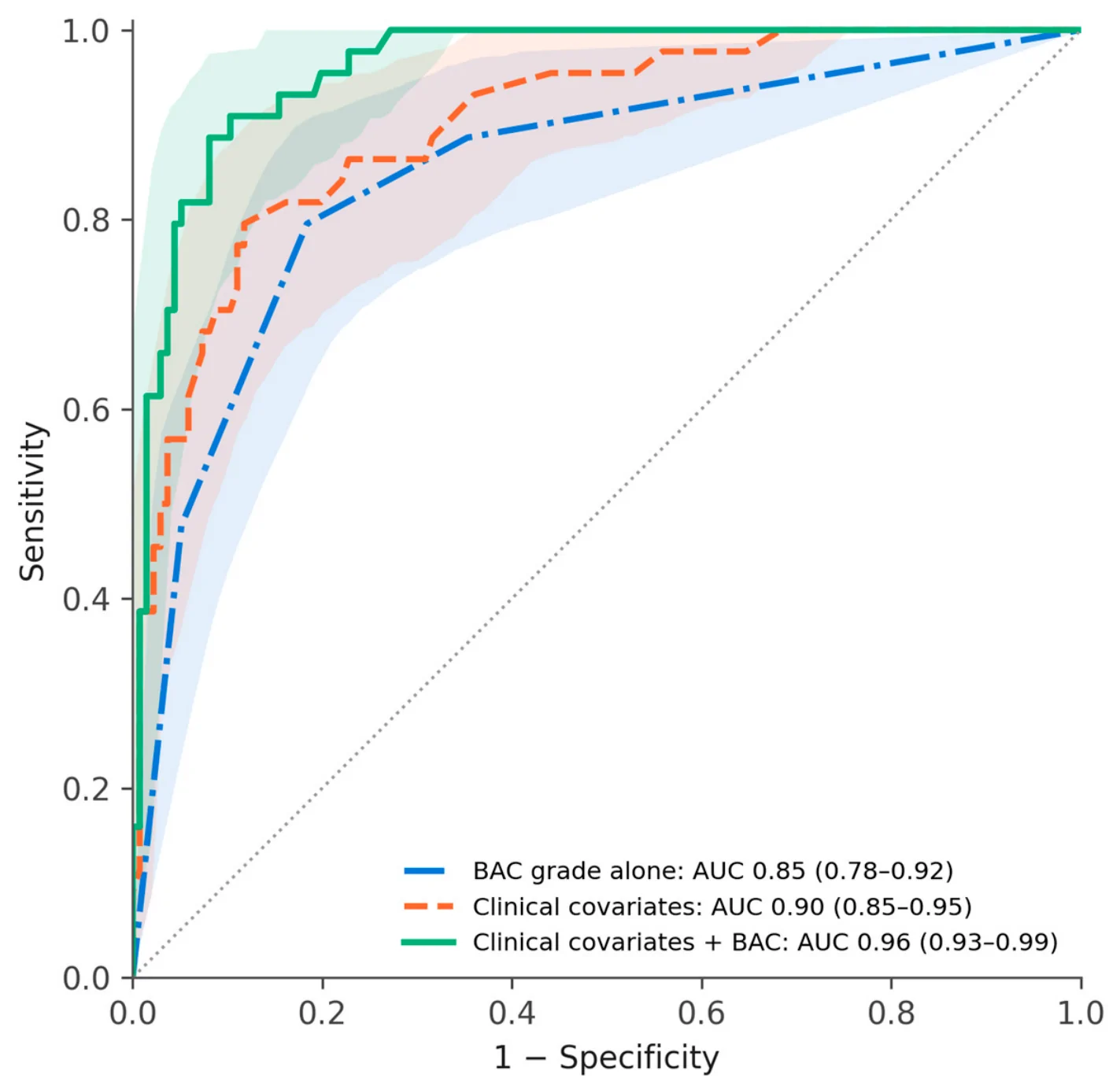
Receiver operating characteristic curves for clinically significant carotid artery stenosis (NASCET grade ≥ 2). Shaded areas indicate bootstrap 95% confidence bands (1000 resamples). Clinical variables were age, diabetes mellitus, hyperlipidemia, hypertension, and smoking. All curves were developed and evaluated in the same 180 women. The dotted diagonal line indicates no discrimination (area under the curve = 0.50).

For clinically significant stenosis, BAC grade alone yielded an AUC of 0.85 (95% CI, 0.78–0.92), whereas the model including BAC grade, diabetes mellitus, and hyperlipidemia yielded an AUC of 0.92 (95% CI, 0.87–0.97; DeLong *p* = 0.007 vs. BAC alone). The clinical model comprising age, diabetes mellitus, hyperlipidemia, hypertension, and smoking had an AUC of 0.90 (95% CI, 0.85–0.95), which increased to 0.96 (95% CI, 0.93–0.99) after addition of BAC grade (DeLong *p* = 0.005) (Table 4, Figure 5). Bootstrap optimism-corrected AUCs were 0.92 for BAC + diabetes mellitus + hyperlipidemia, 0.89 for the clinical model, and 0.95 for the clinical model plus BAC.

Inter-reader agreement for BAC grading was substantial (linearly weighted κ = 0.79; 95% CI, 0.69–0.88). The unweighted κ was 0.67 (95% CI, 0.51–0.80), and exact agreement was observed in 45 of 60 cases (75%). Agreement for the presence versus absence of BAC was complete (κ = 1.00). All disagreements were within one grade and occurred predominantly between grades 2 and 3 (Table S5).

## 4. Discussion

In this clinically selected cohort of postmenopausal women aged 60–65 years who underwent CTA, mammographic BAC was associated with clinically significant carotid artery stenosis. Both the presence and severity of BAC were associated with stenosis, and the association persisted after adjustment for conventional cardiovascular risk factors and across sensitivity analyses.

Previous studies have reported associations between BAC and carotid atherosclerotic findings, including increased carotid IMT and carotid plaque [14,15,16,17]. Our findings are consistent with these observations and extend them by demonstrating an association with carotid luminal stenosis measured by CTA using NASCET criteria. Restricting the cohort to a narrow age range may have reduced age-related confounding, although it also limits generalizability.

The relationship between BAC and coronary artery disease has been studied more extensively. Margolies et al. reported an association between BAC and coronary artery calcification on chest CT, with discriminatory performance similar to conventional risk scores [18]; a meta-analysis of 18 studies likewise found BAC to be associated with coronary artery disease [19]. In contrast, in a SCOT-HEART sub-study of symptomatic women undergoing coronary CTA, BAC was associated with coronary calcification but had poor diagnostic accuracy for coronary artery disease [20]. These findings support the concept that BAC may reflect systemic vascular calcification, but they do not establish equivalence between calcification in different vascular beds, nor do they imply diagnostic value. We did not have coronary imaging data and therefore could not directly assess the relationship between BAC and coronary calcification in this cohort.

After full adjustment, each one-grade increase in BAC was associated with an approximately fourfold increase in the odds of clinically significant stenosis. The wide confidence intervals for binary and category-specific estimates indicate substantial uncertainty in their precise magnitude and reflect the limited number of events. The increase was not uniform at the lower end of the scale: women with grade 1 BAC had only a small, nonsignificant increase in clinically significant stenosis and no severe stenosis, whereas the prevalence rose markedly in grades 2 and 3. Minimal focal BAC may therefore be less specific for systemic atherosclerotic burden than continuous or coarse calcification, although the small subgroup size limits this interpretation.

BAC generally reflects medial arterial calcification (Mönckeberg-type sclerosis), whereas carotid atherosclerosis predominantly involves the intima [5,6]; the two processes are therefore not pathologically equivalent. Shared factors such as age-related vascular change, diabetes mellitus, dyslipidemia, hypertension, and smoking, together with mechanisms such as osteogenic transdifferentiation of vascular smooth-muscle cells, may nevertheless contribute to the observed association [7,8]. Although the association persisted after adjustment for measured risk factors, residual confounding by unmeasured factors, including body mass index, renal function within the normal range, and the duration or control of risk factors, cannot be excluded.

BAC grade alone yielded an AUC of 0.85, and adding BAC to clinical variables significantly improved discrimination; bootstrap validation suggested limited optimism. These findings must nevertheless be interpreted in the context of the selected study population. Because eligibility required clinically indicated CTA, the cohort was enriched for vascular disease: 24.4% had ≥50% stenosis and 10.6% had severe stenosis. This spectrum may inflate apparent discrimination and precludes direct application of predictive values to routine mammography populations, in which carotid stenosis is less prevalent. The discrimination analyses should therefore be regarded as exploratory and do not demonstrate that BAC predicts carotid stenosis or future cerebrovascular events.

The potential clinical relevance of BAC lies in its visibility on an imaging examination that is already routinely performed. Recognition and reporting of BAC may provide an additional opportunity to identify women who could benefit from assessment of modifiable cardiovascular risk factors. BAC alone, however, should not be used as an indication for carotid imaging, and the present findings do not support its use as a screening test for carotid stenosis.

Deep-learning algorithms can automatically detect and quantify BAC on mammograms [4], potentially reducing the limited reproducibility of visual grading, particularly between moderate and severe calcification, and facilitating systematic reporting without additional radiologist workload. Prospective studies in unselected screening populations that combine quantitative BAC measures with standardized carotid imaging and comprehensive risk-factor assessment are needed to determine whether BAC adds information to established risk models, to define clinically meaningful thresholds, and to evaluate whether reporting affects risk-factor management or outcomes. Longitudinal studies should also examine the relationship of BAC progression with carotid atherosclerosis progression and incident cerebrovascular events.

In addition to luminal stenosis, CTA permits characterization of carotid plaque calcification. Spotty calcifications and microcalcifications have been associated with plaque vulnerability, whereas larger nodular calcifications are more often seen in stable plaques. Because the present study was designed to evaluate the relationship between mammographic BAC and luminal stenosis, plaque composition and calcification morphology were not systematically assessed. Future studies could examine whether BAC is associated not only with stenosis severity but also with specific carotid plaque phenotypes.

Strengths of this study include the narrow age range, which reduces the marked age-related confounding that affects studies spanning broader age groups; the use of cervical CTA with NASCET-based luminal stenosis assessment rather than surrogate measures such as carotid IMT; blinded assessment of mammography and CTA by two experienced radiologists; adjustment for major cardiovascular risk factors; and the consistency of the association across alternative BAC definitions and interval-based sensitivity analyses.

This study has several limitations. Because inclusion required clinically indicated CTA and patients were referred for nonspecific symptoms, the study population was a selected, disease-enriched referral cohort rather than a screening population, and selection bias cannot be excluded. The retrospective, single-center design, limited number of outcome events, and restriction to women aged 60–65 years limit precision and generalizability. Individual CTA indications, body mass index, lipid subfractions, and detailed renal-function data were not available, leaving the possibility of residual confounding. Mammography and CTA were not performed simultaneously (median interval, 131 days), although sensitivity analyses suggested that the association was not materially affected by the interval. Inter-reader reproducibility of BAC grading was assessed in a 60-patient subset rather than in the entire cohort, and agreement between grades 2 and 3 was lower than agreement for BAC presence versus absence. In addition, the second reader had evaluated the CTA examinations of the same women approximately 8 months earlier; although the CTA findings were not available during re-grading, recall bias cannot be completely excluded. Finally, the discrimination models were developed and evaluated in the same sample and underwent internal validation only; external prospective validation in population-based cohorts is required.

## 5. Conclusions

In selected postmenopausal women aged 60–65 years who underwent both mammography and clinically indicated CTA, BAC was associated with carotid artery stenosis independently of conventional cardiovascular risk factors. These findings do not establish BAC as a screening or prediction tool for carotid stenosis. Prospective, population-based studies with broader confounder assessment and external validation are needed before any clinical screening application can be considered.

## Figures and Tables

**Table 1:** Characteristics of the study population according to the presence of clinically significant carotid stenosis (*n* = 180).

Characteristic	Overall (*n* = 180)	Stenosis < 50% (*n* = 136)	Stenosis ≥ 50% (*n* = 44)	SMD	*p*
Age, years, mean ± SD	62.1 ± 1.8	61.8 ± 1.7	62.9 ± 1.8	0.61	<0.001
Mammography–CTA interval, days, median (IQR)	131 (75–217)	136 (78–217)	124 (73–218)	−0.05	0.807
Diabetes mellitus, *n* (%)	33 (18.3)	15 (11.0)	18 (40.9)	0.72	<0.001
Hyperlipidemia, *n* (%)	49 (27.2)	20 (14.7)	29 (65.9)	1.22	<0.001
Hypertension, *n* (%)	59 (32.8)	35 (25.7)	24 (54.5)	0.61	<0.001
Smoking, *n* (%)	68 (37.8)	40 (29.4)	28 (63.6)	0.73	<0.001
BAC grade 0, *n* (%)	93 (51.7)	88 (64.7)	5 (11.4)		
BAC grade 1, *n* (%)	27 (15.0)	23 (16.9)	4 (9.1)		
BAC grade 2, *n* (%)	32 (17.8)	18 (13.2)	14 (31.8)		
BAC grade 3, *n* (%)	28 (15.6)	7 (5.1)	21 (47.7)		

Values are mean ± SD, median (IQR), or *n* (%). SMD, standardized mean difference; CTA, computed tomography angiography; BAC, breast arterial calcification; IQR, interquartile range; SD, standard deviation. *p* values were obtained using the Mann–Whitney U test, chi-square test, or Fisher’s exact test, as appropriate.

**Table 2:** Cross-tabulation of BAC grade and carotid stenosis (*n*, column %).

BAC Grade	No Stenosis	Grade 1 (<50%)	Grade 2 (50–69%)	Grade 3 (≥70%)	Total
0—none	67 (76.1)	21 (43.8)	4 (16.0)	1 (5.3)	93
1—mild	20 (22.7)	3 (6.3)	4 (16.0)	0 (0.0)	27
2—moderate	1 (1.1)	17 (35.4)	7 (28.0)	7 (36.8)	32
3—severe	0 (0.0)	7 (14.6)	10 (40.0)	11 (57.9)	28
Total	88	48	25	19	180

**Table 3:** Association between BAC and clinically significant carotid stenosis (≥50%): unadjusted and adjusted odds ratios (*n* = 180; 44 events).

BAC Definition	Unadjusted OR (95% CI)	*p*	Model 1 aOR (95% CI)	*p*	Model 2 aOR (95% CI)	*p*
BAC grade, per grade	3.81 (2.63–5.85)	<0.001	3.44 (2.24–5.66)	<0.001	4.23 (2.50–8.03)	<0.001
BAC grade (categorical)						
Grade 0	Reference		Reference		Reference	
Grade 1	3.08 (0.77–11.75)	0.081	3.40 (0.72–15.67)	0.099	3.58 (0.65–20.20)	0.125
Grade 2	12.61 (4.40–41.16)	<0.001	7.06 (2.17–25.71)	<0.001	10.55 (2.74–49.40)	<0.001
Grade 3	46.13 (14.98–168.09)	<0.001	40.05 (10.73–185.64)	<0.001	49.68 (11.25–294.27)	<0.001
BAC presence (grades 1–3 vs. 0)	14.30 (5.74–43.63)	<0.001	11.51 (4.07–39.58)	<0.001	16.70 (4.99–73.27)	<0.001
BAC severity (grades 2–3 vs. 0–1)	17.27 (7.66–42.60)	<0.001	11.33 (4.48–31.35)	<0.001	18.49 (5.93–70.46)	<0.001
Ordinal model (4-level stenosis), per BAC grade	4.01 (2.93–5.49)	<0.001	—		3.71 (2.69–5.14)	<0.001

Model 1 was adjusted for diabetes mellitus and hyperlipidemia. Model 2 was adjusted for age, diabetes mellitus, hyperlipidemia, hypertension, and smoking. Firth penalized likelihood was used for categorical models. Ordinal model: proportional-odds logistic regression with four-level stenosis grade as the outcome. OR, odds ratio; aOR, adjusted odds ratio; CI, confidence interval.

**Table 4:** Discrimination for clinically significant carotid stenosis: DeLong comparisons and bootstrap internal validation (*n* = 180).

Model	AUC (95% CI)	Optimism-Corrected AUC
BAC grade alone	0.85 (0.78–0.92)	—
DM + HL	0.82 (0.75–0.89)	—
BAC grade + DM + HL	0.92 (0.87–0.97)	0.92
Clinical variables	0.90 (0.85–0.95)	0.89
Clinical variables + BAC grade	0.96 (0.93–0.99)	0.95
Comparison	ΔAUC	DeLong *p*
BAC alone vs. BAC + DM + HL	+0.07	0.007
DM + HL vs. BAC + DM + HL	+0.10	0.001
Clinical variables vs. clinical variables + BAC	+0.06	0.005

Clinical variables: age, diabetes mellitus (DM), hyperlipidemia (HL), hypertension, and smoking. Optimism correction was based on 1000 bootstrap resamples. All estimates were derived from a clinically selected CTA-referred cohort and are exploratory. AUC, area under the curve.

## Data Availability

The data supporting the findings of this study are available from the corresponding author upon reasonable request.

## Supplementary Materials

The following supporting information can be downloaded at https://www.mdpi.com/article/10.3390/jcm15207844/s1, Table S1: 2 × 2 tables for the binary BAC definitions and clinically significant carotid stenosis (n = 180); Table S2: Fully adjusted multivariable logistic regression model for clinically significant carotid stenosis (Model 2; n = 180; 44 events); Table S3: Association between BAC and severe carotid stenosis (≥70%; 19 events); Table S4: Sensitivity analyses for the interval between mammography and CTA; Table S5: Inter-reader agreement for BAC grading (n = 60; stratified random sample, 15 women per BAC grade); Table S6: Characteristics according to BAC grade (n = 180).

## Abbreviations

The following abbreviations are used in this manuscript:

AUC	Area under the curve
BAC	Breast arterial calcification
CI	Confidence interval
CTA	Computed tomography angiography
IMT	Intima–media thickness
NASCET	North American Symptomatic Carotid Endarterectomy Trial
OR	Odds ratio
ROC	Receiver operating characteristic
aOR	Adjusted odds ratio
IQR	Interquartile range
SMD	Standardized mean difference
