# Supplementary material for: Association Between Breast Arterial Calcification on Mammography and Carotid Artery Stenosis on CT Angiography: A Retrospective Study in a Selected Cohort of Women Aged 60–65 Years
Source: J Clin Med. 2026 Oct 12;15(20):7844. doi: 10.3390/jcm15207844 (PMC13706212; doi:10.3390/jcm15207844)
Supplement: Supplementary file 1 [file jcm-15-07844-s001.zip › jcm-4603764-supplementary.pdf]

Supplementary Materials

Association Between Breast Arterial Calcification on Mammography and Carotid Artery Stenosis on CT Angiography: A Retrospective Study in a Selected Cohort of Women Aged 60–65 Years

Table S1. 2 × 2 tables for the binary BAC definitions and clinically significant carotid stenosis (n = 180).

	Stenosis <50%	Stenosis ≥50%	Total
BAC absent (Grade 0)	88	5	93
BAC present (Grades 1–3)	48	39	87
BAC Grades 0–1	111	9	120
BAC Grades 2–3	25	35	60
Total	136	44	180

Unadjusted OR: BAC presence 14.30 (95% CI 5.74–43.63); BAC Grades 2–3 vs. 0–1 17.27 (95% CI 7.66–42.60).

Table S2. Fully adjusted multivariable logistic regression model for clinically significant carotid stenosis (Model 2; n = 180; 44 events).

Variable	aOR (95% CI)	p
BAC grade (per grade)	4.23 (2.50–8.03)	<0.001
Age (per year)	1.49 (1.06–2.18)	0.023
Diabetes mellitus	3.65 (0.94–14.80)	0.061
Hyperlipidemia	15.19 (4.65–60.68)	<0.001
Hypertension	5.76 (1.77–21.51)	0.003
Smoking	5.63 (1.79–20.30)	0.003

aOR, adjusted odds ratio; CI, confidence interval.

Table S3. Association between BAC and severe carotid stenosis (≥70%; 19 events).

BAC specification	Unadjusted OR (95% CI)	p	Model 2 aOR (95% CI)	p
BAC grade, per 1-grade increase	4.17 (2.43–8.32)	<0.001	2.68 (1.52–5.47)	<0.001
Grade 1 vs. 0	1.12 (0.01–21.64)	1.000	1.08 (0.01–22.19)	1.000
Grade 2 vs. 0	18.14 (3.72–177.74)	<0.001	8.82 (1.56–92.73)	0.010
Grade 3 vs. 0	40.52 (8.80–391.03)	<0.001	14.39 (2.66–147.84)	0.001
BAC presence	24.00 (4.78–436.77)	<0.001	8.25 (1.73–82.05)	0.004
BAC Grades 2–3 vs. 0–1	51.00 (10.05–931.18)	<0.001	14.30 (3.10–137.38)	<0.001

Model 2: adjusted for age, diabetes mellitus, hyperlipidemia, hypertension and smoking. Categorical and all Model 2 estimates were fitted with Firth's penalized likelihood. Wide confidence intervals reflect the small number of events.

Table S4. Sensitivity analyses for the interval between mammography and CTA.

Analysis	n	Events	aOR per BAC grade (95% CI)	p
Main analysis (Model 2)	180	44	4.23 (2.50–8.03)	<0.001
Additionally adjusted for interval	180	44	4.29 (2.52–8.28)	<0.001
Interval ≤180 days	109	27	4.68 (2.42–11.78)	<0.001
Interval ≤90 days	59	15	3.32 (1.52–10.63)	<0.001

All models adjusted for age, diabetes mellitus, hyperlipidemia, hypertension and smoking; restricted analyses were fitted with Firth's penalized likelihood. BAC × interval interaction: p = 0.18. Median interval 131 days (IQR 75–217; range 23–326).

Table S5. Inter-reader agreement for BAC grading (n=60; stratified random sample, 15 women per BAC grade).

Reader 1	Reader 2: grade 0	Reader 2: grade 1	Reader 2: grade 2	Reader 2: grade 3	Total
Grade 0	15	0	0	0	15
Grade 1	0	13	2	0	15
Grade 2	0	3	10	2	15
Grade 3	0	0	8	7	15

Reader 1	Reader 2: grade 0	Reader 2: grade 1	Reader 2: grade 2	Reader 2: grade 3	Total
Total	15	16	20	9	60

Reader 1: breast radiologist with 12 years of experience (original assessment). Reader 2: radiologist with 16 years of experience, re-grading approximately 8 months later without access to the original grades, CTA findings or clinical data. Exact agreement 75.0%; agreement within one grade 100%. Unweighted κ 0.67 (95% CI 0.51–0.80); linearly weighted κ 0.79 (0.69–0.88); quadratically weighted κ 0.89 (0.83–0.94). Presence vs. absence of BAC: κ 1.00; Grades 2–3 vs. 0–1: κ 0.83. 95% CIs from 2000 bootstrap resamples.

Table S6. Characteristics according to BAC grade (n = 180).

Characteristic	Grade 0	Grade 1	Grade 2	Grade 3	p
n	93	27	32	28	
Age, years, mean ± SD	61.9 ± 1.7	61.5 ± 1.6	62.5 ± 1.8	62.7 ± 1.8	0.030
Interval, days, median (IQR)	124 (64–234)	176 (122–210)	152 (86–236)	116 (76–183)	0.411
Diabetes mellitus, n (%)	10 (10.8)	3 (11.1)	11 (34.4)	9 (32.1)	0.004
Hyperlipidemia, n (%)	15 (16.1)	5 (18.5)	14 (43.8)	15 (53.6)	<0.001
Hypertension, n (%)	29 (31.2)	7 (25.9)	10 (31.2)	13 (46.4)	0.379
Smoking, n (%)	29 (31.2)	13 (48.1)	11 (34.4)	15 (53.6)	0.108
Stenosis: none, n (%)	67 (72.0)	20 (74.1)	1 (3.1)	0 (0.0)	<0.001
Stenosis: <50%, n (%)	21 (22.6)	3 (11.1)	17 (53.1)	7 (25.0)	
Stenosis: 50–69%, n (%)	4 (4.3)	4 (14.8)	7 (21.9)	10 (35.7)	
Stenosis: ≥70%, n (%)	1 (1.1)	0 (0.0)	7 (21.9)	11 (39.3)	

p values from the Kruskal–Wallis and chi-square tests; p for stenosis: Cochran–Armitage trend test for stenosis ≥50%.
